# Autonomic features of craniofacial neuralgias: a systematic review with meta-analysis

**DOI:** 10.22514/jofph.2024.023

**Published:** 2024-09-12

**Authors:** Davis C Thomas, Priyanka Kodaganallur Pitchumani, Abdul Basir Barmak, Sandeep Talluri, Weiran Jiang

**Affiliations:** ^1^Center for Temporomandibular Disorders and Orofacial Pain, Department of Diagnostic Sciences, Rutgers School of Dental Medicine, Newark, NJ 07102, USA; ^2^Department of Periodontics, University of Iowa College of Dentistry and Dental Clinics, Iowa City, IA 52241, USA; ^3^Clinical Research and Biostatistics, Eastman Institute of Oral Health, Rochester, NY 14614, USA; ^4^Smile Accent, Hereford, TX 79045, USA; ^5^Light Dental Studios, Puyallup, WA 98371, USA

**Keywords:** Neuralgia, Craniofacial neuralgia, Trigeminal neuralgia, Autonomic, Neuralgia management

## Abstract

The aim of the study is to describe the severity, temporal characteristics, and types of autonomic features as they relate to the characteristics of pain of the neuralgias. Also, to describe, based on literature, how these autonomic features can affect the treatment outcomes of patients with craniofacial neuralgias. We carried out a literature search using five databases, PubMed, Embase, OVID, Scopus and Web of Science. The search was executed in accordance with Preferred Reporting Items for Systematic Reviews and Meta-Analyses (PRISMA). The protocol for this systematic review and meta-analysis was registered on PROSPERO CRD42021235319. 40% of all patients with craniofacial neuralgias had at least one autonomic feature. Out of the craniofacial neuralgias, trigeminal neuralgia was the most reported, with lacrimation being the most prevalent concomitant autonomic feature. There was also differences in the occurrence of the autonomic features dependent on which branch of a nerve such as the trigeminal nerve, was afflicted. When trigeminal neuralgia is excluded, the rest of the craniofacial neuralgias had reported autonomic features 28% of the pain events. (95% Confidence Interval: 2–90%). Contrary to the conventional belief, we found certain autonomic features to be more predominant than others, in specific craniofacial neuralgias. The prevalence of the autonomic features for all craniofacial neuralgias in the descending order is as follows, lacrimation, conjunctival injection, nasal congestion, rhinorrhea, flushing, edema/swelling, salivation, ptosis and sweating. With trigeminal neuralgia, the most common autonomic feature was lacrimation, and the least common was nasal congestion.

## 1. Introduction

Neuralgia is defined by the International Association for the Study of Pain (IASP) as pain in the distribution of the nerves. The IASP classification of chronic pain for International Classification of Diseases (ICD-11) published in 2019 refers to trigeminal neuralgia and “other cranial and regional neuralgias and neuropathies”, classified under “chronic neuropathic orofacial pain”. This document also refers to the International Classification of Headache Disorders (ICHD-3) for definitions, subtypes, and sub forms of trigeminal neuralgia and other less frequent cranial neuralgias [1]. Various craniofacial neuralgias are classified under “painful lesions of the cranial nerves” by the International Headache Society (IHS) [2]. The International Classification of Orofacial Pain (ICOP) has also followed the ICHD-3 classification [3]. Craniofacial neuralgias are classically described as paroxysmal entities with unilateral location of symptoms, repeated attacks, shock like/electric quality of pain and the presence of triggers [4]. Autonomic and motor features associated with neuralgias have also been described in the literature [5, 6]. With reference to autonomic features of neuralgias, the current literature is scanty. Craniofacial neuralgias occurring with autonomic features have distinct similarities with other diagnostic entities such as trigeminal autonomic cephalalgias (TAC). It is important for a clinician to differentiate between these two classes, namely craniofacial neuralgias with autonomic features and TACs. The distinction between these two entities may occasionally be difficult. The pathophysiology and the mechanistic principles underlying the presence of autonomic features with craniofacial neuralgias are poorly understood. The primary objective of this study was to find out whether autonomic features accompany craniofacial neuralgias as per the available literature; and if so, what specific autonomic features occur with craniofacial neuralgias. For this, we considered both sympathetic and parasympathetic parameters that represents autonomic features. The secondary objective of the study was to delineate the hierarchy, if any, of the autonomic symptoms that accompany the various craniofacial neuralgias. We also intended to analyze the literature on the possible significance, if any, of the autonomic features as related to the management of craniofacial neuralgias. In addition to autonomic signs and symptoms, we also aimed to explore the presence, the timing, the temporal characteristics, the severity, and the specific type of autonomic features as related to pain characteristics. We looked at all the documented craniofacial neuralgias published in the last three decades, and their association with various autonomic features.

A general search in the literature looking for autonomic features of craniofacial neuralgias revealed a few important findings. The first is that there is a lack of robust literature regarding the topic. Further, there were no systematic reviews or meta-analysis on the specific topic of autonomic features associated with craniofacial neuralgias. A good majority of the published literature on the topic deals with case reports. There were also a few prospective cohort studies. Also, amongst the craniofacial neuralgias, the trigeminal neuralgia was the most published one, followed by a disproportionately lesser number of other craniofacial neuralgias with autonomic features.

When considering autonomic features, apart from the more commonly worked up features such as tearing, rhinorrhea, salivation, there were other features such as increase in blood pressure, heart rate, and respiratory rate [7, 8]. The significance, if any, of the autonomic features as related to the severity of the pain, the chronicity of the condition, or the resistance to therapy were mentioned in isolation in certain individual studies and reports only [9, 10, 11]. To the best of our knowledge and search, this is the first systematic review of its kind looking at the association of autonomic features with craniofacial neuralgias. As alluded to earlier, there were only very few, if any, possible hypotheses on the mechanism of autonomic features and its relation to the chronicity, severity, and duration of the pain attacks. In the current review, we look at the autonomic features as related to the pain parameters, both in a qualitative and quantitative manner. We also explored the incidence and occurrence rate of specific types of autonomic features that present with craniofacial neuralgias. The other factors we looked at, were the association of specific autonomic features with the intensity of the pain. Further, we also looked at what specific neuralgia is most or least associated with autonomic features; and further, if the specific neuralgia is associated with autonomic features, what specific autonomic features were associated with this entity.

## 2. Methods

The protocol for this systematic review and meta-analysis was registered on PROSPERO CRD42021235319. The search was executed in accordance with Preferred Reporting Items for Systematic Reviews and Meta-Analyses (PRISMA) [12].

### 2.1 Search strategy

We carried out a literature search using five databases, PubMed, Embase, OVID, Scopus and Web of Science. The search frame was from 01 January 1990 to 29 February 2024. Our research questions were “What does the literature say about the presence of autonomic features in various craniofacial neuralgias? What is the incidence of specific type of autonomic features in craniofacial neuralgias? Which specific craniofacial neuralgia is most/least associated with autonomic features?” Several keywords and combinations pertinent to the expected craniofacial autonomic features were used in this search including, but not limited to, “autonomic symptoms: salivation, lacrimation, tearing, sweating, rhinorrhea, flushing, nasal congestion and ptosis”. The same strategy was used to search for the cranio-facial neuralgias. These terms included but not limited to trigeminal neuralgia, glossopharyngeal neuralgia, supra-trochlear neuralgia, supraorbital neuralgia, nervus intermedius, and occipital neuralgia. Keywords used are listed in Table 1, and Medical Subject Headings (MeSH) terms are listed in **Supplementary Table 1**. In addition, the reference lists of reviews retrieved during the search were hand-searched to find potentially eligible records.

**Table 1.** Search strategy: keywords for searching.

Concept	Keywords
1. Autonomic features	conjunctival injection OR ptosis OR miosis OR mydriasis OR tearing OR lacrimation OR congestion OR rhinorrhea OR salivation OR flushing OR sweating OR hyperhidrosis OR swelling OR edema OR red OR redness OR autonomic OR autonomic symptoms OR sympathetic OR parasympathetic
2. Diagnosed types of craniofacial neuralgias	sphenopalatine neuralgia OR spheno-palatine neuralgia OR facial neuralgias OR facial neuralgia OR cranial neuralgia OR cranial neuralgias OR trigeminal neuralgia OR occipital neuralgia OR C2–C3 neuralgia OR greater occipital neuralgia OR lesser occipital neuralgia OR glossopharyngeal neuralgia OR glosso-pharyngeal neuralgia OR supra-trochlear neuralgia OR supratrochlear neuralgia OR supra trochlear neuralgia OR supra-orbital neuralgia OR supra orbital neuralgia OR supraorbital neuralgia OR infratrochlear neuralgia OR infra trochlear neuralgia OR infra-trochlear neuralgia OR infra orbital neuralgia OR infraorbital neuralgia OR infra-orbital neuralgia OR nervus intermedius neuralgia OR nervous intermedius neuralgia OR auriculotemporal neuralgia OR intermediate nerve of Wrisberg neuralgia OR great auricular neuralgia OR greater auricular neuralgia OR auricular neuralgia OR lacrimal neuralgia

*Search combines with 1 and 2.*

### 2.2 Inclusion and exclusion criteria

The inclusion criteria were (a) original human studies, (b) Randomized Controlled Trials (RCTs), cohort studies, cross sectional, case-control studies, case reports and case series, (c) studies reporting patients with clear diagnosis of cranio-facial neuralgias following International Association for the Study of Pain (IASP), and International Headache Society (IHS-3) criteria, (d) studies reporting autonomic features as outcome. We selected only articles in English, with access to the complete manuscript. Exclusion criteria were as follows: in order to eliminate the possibility of disease entities that could mimic neuralgias with autonomic features, we excluded trigeminal autonomic cephalalgias, Short-lasting, Unilateral, Neuralgiform headache attacks with Conjunctival injection and Tearing (SUNCT), Short-lasting unilateral neuralgiform headache attacks with cranial autonomic symptoms (SUNA), cluster headache, Frey’s syndrome and other similar entities. *In vitro* and *in vivo* studies, historic reviews, commentaries, and letters addressed to the editor were also excluded. If the neuralgia was secondary to another cause, such as trauma or brain tumors, they were excluded. Manuscripts with data regarding other-than-human subjects were excluded.

Duplicated articles were removed, titles and abstracts of the rest of the articles were reviewed by two authors (DCT and WJ) following our established inclusion and exclusion criteria; upon which, the same was approved by ST and PKP. After reviewing the title and abstract, articles that did not address the Population, Intervention, Comparison, Outcome (PICO) questions were eliminated. Articles in their entirety were thoroughly reviewed by DCT and WJ and reconfirmed by ST and PKP. Disagreements, if any, among the authors during screening were resolved via discussion with the corresponding author, DCT.

### 2.3 Data extraction

DCT, WJ, PKP and ST independently extracted the following data from eligible studies: (1) titles, (2) authors, (3) year of publication, (4) study design, (5) sample size, (6) types of craniofacial neuralgia, (7) number of patients with at least one autonomic feature, including conjunctival injection, ptosis, lacrimation, sinus congestion, rhinorrhea, salivation, flushing, sweating and “other” autonomic features (redness of the eyelid, persistent edema, facial swelling, malar swelling), (8) temporal characteristics of the pain, and (9) intensity, as it corresponds to the autonomic features.

### 2.4 Quality assessment for the included studies

Joanna Brigg’s institute (JBI) risk of bias tool was used to estimate overall bias in the included studies [13]. AB and ST assessed the quality of the included studies. As the included studies are case reports, case series, cross-sectional and cohort studies, a separate list of questions were used to estimate the overall quality of the included studies (Table 2). The quality of the studies was designated as low, moderate and high, according to the JBI risk of bias tool. A corresponding traffic light plot was created for the results.

**Table 2.** Domains for quality assessment.

Study type	Domains
Case report	
	D1- Were patients’ demographic characteristics clearly described?
	D2- Was the patient’s history clearly described and presented as a timeline?
	D3- Was the current clinical condition of the patient on presentation clearly described?
	D4- Were diagnostic tests or assessment methods and the results clearly described?
	D5- Was the intervention(s) or treatment procedure(s) clearly described?
	D6- Was the post-intervention clinical condition clearly described?
	D7- Were adverse events (harms) or unanticipated events identified and described?
	D8- Does the case report provide takeaway lessons?
Case series	
	D1- Were there clear criteria for inclusion in the case series?
	D2- Was the condition measured in a standard, reliable way for all participants included in the case series?
	D3- Were valid methods used for identification of the condition for all participants included in the case series?
	D4- Did the case series have consecutive inclusion of participants?
	D5- Did the case series have complete inclusion of participants?
	D6- Was there clear reporting of the demographics of the participants in the study?
	D7- Was there clear reporting of clinical information of the participants?
	D8- Were the outcomes or follow-up results of cases clearly reported?
	D9- Was there clear reporting of the presenting site(s)/clinic(s) demographic information?
	D10- Was statistical analysis appropriate?
Cross-sectional study	
	D1- Were the criteria for inclusion in the sample clearly defined?
	D2- Were the study subjects and the setting described in detail?
	D3- Was the exposure measured in a valid and reliable way?
	D4- Were objective, standard criteria used for measurement of the condition?
	D5- Were confounding factors identified?
	D6- Were strategies to deal with confounding factors stated?
	D7- Were the outcomes measured in a valid and reliable way?
	D8- Was appropriate statistical analysis used?
Cohort study	
	D1- Were the two groups similar and recruited from the same population?
	D2- Were the exposures measured similarly to assign people to both exposed and unexposed groups?
	D3- Was the exposure measured in a valid and reliable way?
	D4- Were confounding factors identified?
	D5- Were strategies to deal with confounding factors stated?
	D6- Were the groups/participants free of the outcome at the start of the study (or at the moment of exposure)?
	D7- Were the outcomes measured in a valid and reliable way?
	D8- Was the follow up time reported and sufficient to be long enough for outcomes to occur?
	D9- Was follow up complete, and if not, were the reasons to loss to follow up described and explored?
	D10- Were strategies to address incomplete follow up utilized?
	D11- Was appropriate statistical analysis used?

### 2.5 Narrative synthesis

A preliminary narrative synthesis utilizing all the included studies was performed. The objective here was to assess the consistency of the association of autonomic features with the individual types of neuralgias, intensity of the pain, and other variables mentioned in these studies. We also took into consideration the methodological quality of these studies. An initial assessment of the incidence and prevalence of autonomic features occurring with craniofacial neuralgias was performed. The data extracted were used to describe the individual studies. Then, the individual autonomic feature/s and their association with specific craniofacial neuralgias were described. The consistency of estimates of the association between each autonomic feature, intensity of pain and type of neuralgia were examined, and their statistical significance of occurrence with the neuralgias were assessed.

### 2.6 Quantitative synthesis—meta-analysis

Two authors AB and ST generated the forest plot for meta-analysis. Any conflict between the authors was resolved with the help of third author WJ. Due to the variations in the studies, demographics, location and authors, a random effects model was adopted. Random effects models of meta-analyses were utilized on all included studies to quantify the incidence of autonomic features in craniofacial neuralgias. Forest plots were constructed to visualize heterogeneity between the studies. Subgroup analyses were performed based on specific autonomic features and type of neuralgias, utilizing random effects models, depending on the heterogeneity of the included studies. All statistical analyses were performed using R 1.4.

## 3. Results

### 3.1 Study characteristics

The initial online search included a total of 3447 articles (1321 PubMed, 624 Embase, 358 OVID, 410 Web of science and 734 Scopus). Of these 3447 articles, 1515 were duplicates and were removed. After screening the titles and abstracts, 1892 articles did not fit the inclusion criteria, therefore were excluded. The full-text articles of the remaining 40 studies were obtained, and thoroughly evaluated by 3 independent authors (DCT, ST and WJ). Any uncertainties or disagreements were resolved by discussion with the other authors (PKP and AB). In total, 18 articles were excluded (no positive or negative reported incidence of autonomic features (n = 4); no specific data on autonomic features (n = 7); no clear diagnosis of craniofacial neuralgias (n = 4); autonomic features were caused by reasons other than neuralgias (n = 2), article shared/replicated the same data as another article that was already included in the study (n = 1)). As a result, a total of 22 articles [6, 9, 10, 11, 14, 15, 16, 17, 18, 19, 20, 21, 22, 23, 24, 25, 26, 27, 28, 29, 30, 31] were included for the systematic review and meta-analysis (Fig. 1).

**Fig. 1.** PRISMA flowchart.

11 case reports, three case series, five cross-sectional and three cohort studies were included. A total of 935 participants were identified. Number of patients with at least one autonomic symptom was 382. Sweating was reported in five articles [19, 23, 28, 30, 31], and a total of 11 patients out of 284, had this autonomic feature. Nasal congestion was reported in eight articles [6, 10, 11, 19, 23, 25, 28, 30], and a total of 26 patients out of 169, had this autonomic feature. Salivation was reported in six articles [9, 17, 19, 23, 28, 30], and a total of 61 patients out of 601, had this autonomic feature. Rhinorrhea was reported in 12 articles [9, 10, 11, 19, 21, 23, 24, 25, 28, 29, 30, 31], and a total of 117 patients out of 884, had this autonomic feature. Ptosis was reported in eight articles [18, 19, 21, 23, 24, 28, 30, 31], and a total of 28 patients out of 764, had this autonomic feature. Lacrimation was reported in 17 articles [6, 9, 10, 11, 15, 17, 19, 20, 21, 22, 23, 24, 25, 26, 28, 29, 30], and a total of 208 patients out of 764, had this autonomic feature. Flushing was reported in seven articles [9, 19, 21, 23, 28, 29, 30], and a total of 72 patients out of 682, had this autonomic feature. Conjunctival injection was reported in 11 articles [10, 11, 18, 19, 21, 22, 23, 24, 28, 30, 31], and a total of 59 patients out of 315, had this autonomic feature. Edema/swelling was reported in 7 articles [11, 19, 21, 22, 28, 29, 30], and a total of 22 patients out of 209, had this autonomic feature. Trigeminal neuralgia was reported in 11 articles [9, 10, 11, 19, 21, 24, 25, 26, 29, 30, 31], and a total of 358 patients out of 882, had at least one autonomic feature. Other types of craniofacial neuralgias were reported in 11 articles [6, 14, 15, 16, 17, 18, 20, 22, 23, 27, 28], and a total of 24 patients out of 71, had at least one autonomic feature. Table 3 summarizes the characteristics and findings of the included studies.

**Table 3.** Overview of included studies.

Characteristics of included studies					Number of patients who have										Quality assessment
Author	Year	Study design	Diagnosis of neuralgia	Patients	At least one AS	Sweating	Nasal congestion	Salivation	Rhinorrhea	Ptosis	Lacrimation	Flushing	Conjunctival injection	Edema/swelling	
Rasmussen	1991	Cohort	Trigeminal	474	199	NA	NA	35	51	NA	139	44	NA	NA	Unclear
Bouhassira	1994	Case report	Trigeminal	1	1	0	0	0	1	0	1	0	1	0	Low
Sjaastad	1997	Cross sectional	Trigeminal	19	10	NA	0	NA	2	NA	8	NA	3	NA	Unclear
Benoliel	1998	Cross sectional	Trigeminal	22	6	NA	1	NA	1	NA	6	NA	NA	NA	Unclear
Sesso	2001	Case report	Trigeminal	1	1	NA	0	NA	0	NA	1	NA	1	1	Low
Pareja	2002	Case report	Trigeminal	2	2	NA	NA	NA	0	0	2	NA	2	NA	Low
Sato	2007	Case report	Superior laryngeal	2	1	NA	NA	NA	NA	NA	1	NA	NA	NA	Low
Benoliel	2009	Cohort	Trigeminal	31	7	NA	NA	NA	NA	NA	7	NA	NA	NA	Low
Riederer	2010	Case report	Occipital and nervus intermedius	2	0	NA	NA	NA	NA	NA	NA	NA	NA	NA	Low
Simms	2011	Cross sectional	Trigeminal	92	62	2	0	26	35	20	25	24	17	15	No information
Pareja	2013	Case report	Lacrimal	2	0	NA	NA	NA	NA	NA	0	NA	NA	NA	Low
Molina	2014	Cross sectional	Occipital	32	11	0	24	0	0	0	0	0	0	0	High
Maarbjerg	2014	Cross sectional	Trigeminal	158	48	9	NA	NA	25	7	NA	NA	34	NA	Low
Khan	2015	Case report	Trigeminal	1	1	NA	NA	NA	1	1	1	1	1	1	Low
Haviv	2015	Cohort	Trigeminal	81	21	NA	NA	NA	1	NA	14	3	NA	4	Low
Pareja	2015	Case series	Infratro-chlear	7	7	NA	NA	NA	NA	NA	NA	NA	NA	NA	Low
Pareja	2017	Case series	Supratro-chlear	15	0	NA	NA	NA	NA	NA	NA	NA	NA	NA	Low
Pirillo	2018	Case report	Nervus intermedius	1	0	NA	NA	0	NA	NA	0	NA	NA	NA	High
Villar-Quilles	2018	Case series	Infratro-chlear	7	0	NA	NA	NA	NA	0	NA	NA	0	NA	High
Lee	2019	Case report	Occipital	1	1	NA	NA	NA	NA	NA	1	NA	0	1	Low
Onoda	2020	Case report	Nervus intermedius	1	1	0	0	0	0	0	1	0	0	NA	Low
Thomas	2021	Case report	Occipital	1	1	NA	1	NA	NA	NA	1	NA	NA	NA	Low

*AS: Autonomic symptoms; NA: Not applicable.*

### 3.2 Quality assessment

Results of the quality assessment showed that 15 studies were classified as high quality [6, 11, 14, 15, 16, 19, 20, 21, 22, 23, 24, 26, 27, 29, 31], three as low quality [17, 18, 28], three as unclear (citation) [9, 10, 25] and one [30] as “no information” (Table 3). Among the 11 case reports included in this systematic review [6, 11, 14, 15, 17, 19, 20, 21, 22, 23, 24], one [17] study is low in quality, nine (*i.e.*, 90% of the included studies) [6, 11, 14, 15, 19, 20, 21, 22, 23, 24] are of high quality. Among the three studies included in the case-series [16, 18, 27], two [16, 27] studies have high quality, and one [18] has low quality. Among the) five cross-sectional studies [10, 25, 28, 30, 31], one [31] study is of high quality, one [28] study is of low quality, two [10, 25] studies are unclear, and there is no information for one [30] study. Among the three cohort studies included [9, 26, 29], one [9] study is of unclear quality, and two [26, 29] studies are of high quality. The complete quality assessment of individual studies is presented in **Supplementary Tables 1-1,1-2,1-3,1-4** and **Supplementary Figs. 1,2,3,4**.

#### 3.2.1 Heterogeneity analysis

When autonomic features were forest plotted using Random Effects Model (REM), heterogeneity is high and significant in salivation, rhinorrhea and flushing. It is low and insignificant in sweating, conjunctival injection, lacrimation, and edema/swelling. While features like nasal congestion, and ptosis have moderately high heterogeneity, it is insignificant in nasal congestion and significant in ptosis. Overall, heterogeneity of the included studies is moderately high (*I*^2^ = 58%, *p *< 0.01). The proportion of patients with autonomic features is 0.40 (95% CI: 0.26–0.57).

#### 3.2.2 Quality of evidence

The overall quality of the included studies is high in fifteen studies, low in three studies, unclear in three studies, and no information can be obtained from one study; when all the designs including case report, case series and cohort are reconciled. From the plots, it can be inferred that the overall quality of the included studies is high.

### 3.3 Narrative synthesis 

#### 3.3.1 Sweating 

Simms reported sweating in 2.2% of the patients [30]. It is to be noted that this autonomic symptom is the only sympathetic component, meanwhile all other symptoms reported in this study are parasympathetic activation or sympathetic inhibition. Maarbjerg reported sweating in 6% of all the patients with trigeminal neuralgia [31]. The same study reported 19% of Trigeminal Neuralgia (TN) with autonomic features had sweating as an autonomic feature.

#### 3.3.2 Nasal congestion

Molina reported 40.6% of occipital neuralgia patients having nasal congestion, and additional 34.4% with “nasal occlusion” [28]. Thomas *et al*. [6] reported one case of occipital neuralgia involving nasal congestion. Benoliel reported one patient out of 22 TN patients had nasal congestion [25].

#### 3.3.3 Salivation

Rasmussen reported salivation as the least common autonomic feature with trigeminal neuralgia [9]. In general, however, this study reported that when V3 division was involved, salivation was the dominant autonomic symptom. Salivation was present in 47% of the patients with TN with lacrimation. When patients had non-neuralgiform pain, salivation was rare. 7% of patients with “typical TN” had salivation, and 8% of “atypical TN” had salivation. Simms reported salivation as the most common symptom when V3 was involved [30]. Simms also reported that when pain was triggered by swallowing and for patients with excess salivation, microvascular decompression did not improve autonomic symptoms. When V1 was involved, 20% of patients had salivation; 24% for V2, 30% for V3 in patients with pain.

#### 3.3.4 Rhinorrhea 

According to Simms, rhinorrhea was present when V1 division was involved [30]. Rasmussen reported that rhinorrhea was the most common second autonomic symptom in addition to lacrimation, and it was predominant with V2 involvement [9]. He also reported rhinorrhea in 9% of patients with “typical TN” and 13% with “atypical TN”. According to Bouhassira [19] and Sjaastad [10], rhinorrhea was the least common symptom with TN. Sjaastad also proposed that rhinorrhea may be the most singular autonomic phenomena that best differentiated SUNCT from the TN of V1 division. Maarbjerg reported 16% of TN cases with rhinorrhea [31].

#### 3.3.5 Ptosis

According to Simms, ptosis is the second most common autonomic symptom when V1 division is involved [30]. Further, ptosis occurred in 25% of patients with V1 involvement and 19% of patients with V2 and V3 involvement. Maarbjerg reported ptosis in 4% of TN cases [31].

#### 3.3.6 Lacrimation

Sjaastad reported that lacrimation was the most frequent autonomic feature with trigeminal neuralgia of the V1 division [10]. According to Sjaastad, lacrimation occurred during the later stage of the disease, and when the attacks were severe and long-standing. In addition, while general autonomic features were present in approximately 50% of V1 TN cases, lacrimation was present in 100% of the V1 TN cases with autonomic features. Lacrimation as the sole autonomic feature occurred only with a minimum amount of tears, when the attacks were of long duration and maximum severity.

Rasmussen [9], Sjaastad [10], Pareja [24], Benoliel [26], Simms [30], Onoda [23] reported lacrimation as the most common autonomic feature with craniofacial neuralgias. Benoliel is the only one that describes a possible mechanism of lacrimation in craniofacial neuralgias [25]. Rasmussen [9], Benoliel [25], Simms [30] reported lacrimation as being the most common symptom 27% of TN with the involvement of V1 division of the trigeminal nerve. In addition, according to Rasmussen, lacrimation with one or more other autonomic features occurred in 32% of the TN cases [9]. He also reported that, when lacrimation was present, rhinorrhea was the most common second autonomic symptom in 90% of the patients. Pareja reported lacrimation associated with increased severity of attacks, and increased chronicity of the pain [24]. Sato reported lacrimation to be associated with more intense pain [20]. Benoliel reported lacrimation was the most common feature associated with pain-related awakening from sleep [26]. He also reported lacrimation associated with the longest duration of disease history, but no difference in pain intensity or duration among patients with and without lacrimation. Onoda reported lacrimation associated with nervus intermedius neuralgia [23]. Riederer also reported no lacrimation with occipital and nervus intermedius neuralgias [14]. Thomas reported ipsilateral lacrimation with occipital neuralgia [6]. Sesso reported severe and long term attacks brought about lacrimation [11]. Out of the studies we looked at, Pareja [15], Molina [28], Pirillo [17] reported no association with lacrimation for the craniofacial neuralgias they were looking at. According to Haviv, lacrimation was associated with approximately 20% of patients with trigeminal neuralgia [29].

#### 3.3.7 Flushing

Rasmussen reported that flushing was a predominant autonomic symptom when V2 division only was involved [9]. Further, 62% of patients with lacrimation showed flushing. Simms reported flushing was involved with 26% of the trigeminal neuralgia cases [30]. Haviv reported flushing with 3.2% of TN cases when the pain was less than 2 minutes, and 5% of TN cases when the pain lasted more than 2 minutes [29].

#### 3.3.8 Conjunctival injection

Sjaastad [10] and Simms [30] reported conjunctival injection to be the second most common autonomic feature with neuralgias. Pareja reported that the finding of “mild conjunctival injection” was not clear due to confounding factors [24]. Sesso reported conjunctival injection to be associated with severe pain attacks and long duration of pain [11]. Riederer reported that occipital and nervus intermedius neuralgias are not accompanied by conjunctival injection [14]. Simms also reported that conjunctival injection is mostly associated with the V1 involvement [30].

#### 3.3.9 Edema and swelling

Several authors reported edema and /or swelling related to the pain episodes. Sesso reported 1 patient with red eyelid and persistent edema [11]. Simms reported 15 out 92 patients with TN having facial swelling (edema) [30]. Khan reported a case with TN with periorbital edema [21]. Haviv had reported 4 out of 81 TN cases having edema [29]. Lee reported 1 patient with occipital neuralgia with malar swelling (edema) [22].

### 3.4 Meta-analysis 

382 patients had autonomic symptoms out of 953 patients included from all the articles. Five [14, 15, 16, 17, 18] studies which stated that the patients do not have any autonomic symptoms were included in the study as a negative group. Heterogeneity of the included studies is moderately high (*I*^2^ = 54%, *p *< 0.01). The proportion of patients with autonomic features is 0.42 (95% CI: 0.25–0.61) (Fig. 2).

**Fig. 2.** Forest plot: association of autonomic symptoms and craniofacial neuralgias. CI: confidence interval.

Subgroup analysis was done on the various autonomic features reported in the articles. Autonomic features on which the subgroup analysis was performed include, sweating, nasal congestion, salivation, rhinorrhea, ptosis, lacrimation, conjunctival injection, and flushing. Subgroup analysis was performed comparing trigeminal neuralgia with other types of craniofacial neuralgias, which include lacrimal, superior laryngeal, occipital, infra trochlear, supra trochlear, and nervus intermedius neuralgias.

#### 3.4.1 Meta-analysis: sweating and craniofacial neuralgias

Only five [19, 23, 28, 30, 31] studies reported patients with sweating. Out of a total of 284 patients included in these studies, eleven were reported to have shown sweating. The heterogeneity (*I*^2^ = 0%, *p* = 0.81) is low and insignificant. The proportion of patients with sweating is 0.04 (95% CI: 0.01–0.09) (Fig. 3).

**Fig. 3.** Forest plot: association of sweating with craniofacial neuralgias. CI: confidence interval.

#### 3.4.2 Meta-analysis: nasal congestion and craniofacial neuralgias

Eight studies [6, 10, 11, 19, 23, 25, 28, 30] reported patients with nasal congestion. 26 patients had nasal congestion out of a total of 169 patients included in the studies. The heterogeneity (*I*^2^ = 50%, *p* = 0.05) is moderately high and insignificant. The proportion of patients with nasal congestion is 0.02 (95% CI: 0.0–0.57) (Fig. 4).

**Fig. 4.** Forest plot: association of nasal congestion and craniofacial neuralgias. CI: confidence interval.

#### 3.4.3 Meta-analysis: Salivation and craniofacial neuralgias

Six articles [9, 17, 19, 23, 28, 30] reported patients with salivation as an autonomic symptom. 61 patients reported salivation out of a total of 601 patients included in all the six studies together. The proportion of patients with salivation is 0.06 (95% CI: 0.01–0.31) using REM. There is a high and significant heterogeneity (*I*^2^ = 83%, *p* < 0.01) in the included studies (Fig. 5).

**Fig. 5.** Forest plot: association of salivation and craniofacial neuralgias. CI: confidence interval.

#### 3.4.4 Meta-analysis: rhinorrhea and craniofacial neuralgias

Out of a total of 884 patients included in 12 studies [9, 10, 11, 19, 21, 23, 24, 25, 28, 29, 30, 31], 117 patients had rhinorrhea as an autonomic symptom. The proportion of patients who had rhinorrhea is 0.09 (95% CI: 0.03–0.23) using REM. There is a high and significant heterogeneity (*I*^2^ = 77%, *p* < 0.01) in the included studies (Fig. 6).

**Fig. 6.** Forest plot: association of rhinorrhea and craniofacial neuralgias. CI: confidence interval.

#### 3.4.5 Meta-analysis: ptosis and craniofacial neuralgias

Eight studies [18, 19, 21, 23, 24, 28, 30, 31] reported ptosis as a symptom occurring with craniofacial neuralgias. Among a total of 294 patients included in these eight studies, 28 patients reported ptosis. The proportion of patients who have ptosis is 0.05 (95% CI: 0.01–0.25). Heterogeneity is moderately high and significant (*I*^2^ = 53%, *p* = 0.04). A random-effects model was used in this meta-analysis (Fig. 7).

**Fig. 7.** Forest plot: association of ptosis and craniofacial neuralgias. CI: confidence interval.

#### 3.4.6 Meta-analysis: lacrimation and craniofacial neuralgias

208 patients reported lacrimation as a symptom, out of a total number of 764 patients included in the 17 studies together [6, 9, 10, 11, 15, 17, 19, 20, 21, 22, 23, 24, 25, 26, 28, 29, 30]. The proportion of patients who have lacrimation is 0.34 (95% CI: 0.16–0.57) using REM. Heterogeneity is low and insignificant (*I*^2^ = 0%, *p *= 0.96) (Fig. 8).

**Fig. 8.** Forest plot: association of lacrimation and craniofacial neuralgias. CI: confidence interval.

#### 3.4.7 Meta-analysis: flushing and craniofacial neuralgias

Seven studies [9, 19, 21, 23, 28, 29, 30] reported flushing as a symptom with craniofacial neuralgias. 72 patients reported flushing out of a total number of 682 patients included in the studies. The proportion of patients who have flushing is 0.08 (95% CI: 0.02–0.24). Heterogeneity is significant (*I*^2^ = 75%, *p* < 0.01) and the random-effects model was used (Fig. 9).

**Fig. 9.** Forest plot: association of flushing and craniofacial neuralgias. CI: confidence interval.

#### 3.4.8 Meta-analysis: conjunctival injection and craniofacial neuralgias

Eleven articles reported conjunctival injection as a symptom of craniofacial neuralgias [10, 11, 18, 19, 21, 22, 23, 24, 28, 30, 31]. 59 patients reported conjunctival injection out of a total of 315 patients included in the studies. The proportion of patients who have conjunctival injection is 0.24 (95% CI: 0.04–0.68) using REM. Overall heterogeneity (*I*^2^ = 0%, *p*= 1) is low and insignificant (Fig. 10).

**Fig. 10.** Forest plot: association of conjunctival injection and craniofacial neuralgias. CI: confidence interval.

#### 3.4.9 Meta-analysis: edema/swelling and craniofacial neuralgias

Seven articles reported edema/swelling [11, 19, 21, 22, 28, 29, 30]. 22 patients reported edema/swelling out of a total of 209 patients included in the studies. The proportion of patients who had edema/swelling is 0.25 (95% CI: 0.02–0.87) using REM. Overall heterogeneity (*I*^2^ = 0%, *p* = 0.53) is low and insignificant (Fig. 11).

**Fig. 11.** Forest plot: association of edema/swelling and craniofacial neuralgias. CI: confidence interval.

#### 3.4.10 Meta-analysis: comparison of presence of autonomic features in trigeminal neuralgia with those in other types of craniofacial neuralgias

Eleven articles [9, 10, 11, 19, 21, 24, 25, 26, 29, 30, 31] reported patients who had TN with autonomic features. Out of a total of 882 patients with TN, 358 reported at least one autonomic symptom. Eleven articles [6, 14, 15, 16, 17, 18, 20, 22, 23, 27, 28] reported patients who had other types of craniofacial neuralgias. The proportion of patients with TN who had autonomic features is 0.42 (95% CI: 0.30–0.55), and the heterogeneity (*I*^2^ = 78%, *p* < 0.01) is high and significant. For patients with other types of craniofacial neuralgias, proportion of patients with other types of craniofacial neuralgias who had autonomic features is 0.28 (95% CI: 0.02–0.90), and the heterogeneity (*I*^2^ = 0%, *p* = 1) is low and insignificant.

A subgroup analysis was done with trigeminal neuralgia against other types of neuralgias. From the forest plot, we can conclude that 42% of patients had TN with heterogeneity of 78% and the *p*-value is significant. Heterogeneity of other neuralgias is 0% and the *p*-value is 1 and is insignificant (Fig. 12).

**Fig. 12.** Forest plot: comparison of presence of autonomic features of trigeminal neuralgia with those of other types of craniofacial neuralgias. CI: confidence interval.

## 4. Discussion

This systematic review and meta-analysis investigated the incidence of various autonomic features in craniofacial neuralgias. One of the interesting facts we observed upon doing our initial literature search was the non-congruence of terminology we came across in various articles. For example, change of color of the skin (a function of the autonomic nervous system) was variedly reported as redness, flushing and erythematous. It should be noted here that this feature might not be so apparent in individuals with darker skin. Congestion of the nasal passages was reported as nasal congestion or sinus congestion or nasal fullness. In the strict sense, we the authors believe these may be two different clinical entities, nonetheless indicating autonomic activation. Lacrimation and tearing were used interchangeably. It must be noted that it may be difficult to differentiate whether it is the tearing that is spontaneously related to autonomic activation or provoked by the patients rubbing their eyes in response to the intense irritation. In related future studies, it may be prudent to interview the patient as to the automatism of the tearing as opposed to being provoked by the patient. Further, pain of craniofacial neuralgias being typically unilateral, the autonomic feature of the same is expected ipsilaterally. Facial and eyelid swelling were described as edema. Here it must be noted that it is difficult to differentiate a swelling and a perception of swelling (dysesthesia). The assumption when we looked at these articles is the fact that the reporting clinician and the patient actually “saw” and verified the swelling. Rhinorrhea, nasal discharge and runny nose were used synonymously as well.

We found that two manuscripts with relatively good number of subjects, Rasmussen [9] and Maarbjerg [31] reported the results with variation, in the sense that the former had 139 TN patients with lacrimation, with no conjunctival injection reported, while the latter had 34 conjunctival injection and/or lacrimation reported. Similarly, Haviv [29] reported 14 TN patients with lacrimation but none was reported to have conjunctival injection. The interesting thought in these studies is that it is possible to have lacrimation with pain, with absolutely no redness of conjunctiva. The manner in which these features were reported may explain the heterogeneity of the findings.

As per Rasmussen [9], the most common autonomic features that present with trigeminal neuralgia, in the descending order of prevalence, are lacrimation, rhinorrhea, swelling, flushing and salivation. As per Sjaastad [10], the descending order of prevalence of autonomic features with trigeminal neuralgia was lacrimation, conjunctival injection and rhinorrhea. Most of the studies reported lacrimation/tearing as the most common autonomic feature occurring with TN. Interestingly, the most common prevalent autonomic symptom in neuralgias other than TN was also lacrimation.

As per our review/meta-analysis, the prevalence of the autonomic features for all craniofacial neuralgias in the descending order is as follows, lacrimation, conjunctival injection, nasal congestion, rhinorrhea, flushing, edema/swelling, salivation, ptosis and sweating. Also, the prevalence of the autonomic features for trigeminal neuralgia in the descending order is as follows, lacrimation, ​​rhinorrhea, flushing, salivation, conjunctival injection, ptosis, edema/swelling, sweating and nasal congestion. With TN, the most common autonomic feature was lacrimation, and the least common was nasal congestion. In the neuralgias other than cranial (*i.e.*, occipital neuralgia), the most prevalent autonomic symptom was nasal congestion.

Lacrimation is due to (1) dysfunction in the parasympathetic part of the autonomic nervous system, (2) trigeminal autonomic reflex through a link between cranial nerve V and VII at the brainstem level, (3) stimulation of the mucosa of eye and nose through the sensory nervous system, (4) stimulation of the trigeminal ganglion (5) “Tic” of the facial nerve accompanying TN pain (6) several mechanisms acting either separately or together produce lacrimation in TN. Parasympathetic activation can cause salivation in TN cases [25]. V1, V2 involvement had more lacrimation; when only V2 was involved, rhinorrhea, swelling and flushing dominated; when V3 was involved, salivation was the dominant autonomic symptom [9]. Salivation occurs more with the involvement of V3 division of the trigeminal nerve. Lacrimation occurred more with involvement of V2 [25]. Autonomic phenomena occurred more during the later stages, severe attacks, and long-lasting attacks of neuralgias [10]. When autonomic features were involved, there was wider distribution of the pain. Reduction in autonomic symptoms occurred concomitantly with pain reduction [9]. Autonomic features outlasted the pain attacks by a few seconds [19]. The dosage of carbamazepine necessary for adequate analgesia was doubled in TN with lacrimation, compared to TN without lacrimation [25].

More than one autonomic feature occurring was more prevalent than a single autonomic feature in TN patients. Lacrimation with one or more other autonomic features occurred in 32% of the TN cases. Amongst TN with lacrimation, rhinorrhea was the second most common autonomic symptom (90%), followed by swelling and flushing (62%), salivation (47%) [9]. It must be noted that there are literature reports of noxious stimuli caused by trigeminal neuralgia causing peripheral and central nerve sensitization. It has been proposed that poorly controlled trigeminal neuralgia may trigger the onset of SUNCT [32].

One of the limitations of this study is the heterogeneity of the included studies. The reason for this heterogeneity may be multifactorial, including but not limited to, the variation of defining autonomic symptomatology, the non-visibility/non-recording of symptoms, and various terms being used synonymously. It must also be noted that entities such as SUNCT/SUNA may symptomatically mimic TN with autonomic features. We cannot be certain that the diagnosis in the included articles may have reflected this possible overlap. Observations were also made as to the level of prognosis as related to the type and severity of autonomic features. However, it must be noted that these observations are not concluded from statistical methods; they reflect what was reported in the selected articles. Further, the articles included have certain differences in data collection methods and study designs. Out of 22 studies included in this analysis, nine were case reports and three were case series studies. The data from this may have contributed to the heterogeneity of the study. Future prospective, well controlled, well-defined studies are necessary to better elucidate the results from more succinct and relevant data. Also, succinct management/medication protocols should be developed guided by the presence or absence, chronicity, and the intensity of the autonomic features in these craniofacial neuralgias.

## 5. Conclusion

This is the first of its kind, reviewing and analyzing the autonomic symptoms that accompany craniofacial neuralgias. Contrary to the conventional belief, we found certain autonomic features to be more predominant than others, in specific craniofacial neuralgias. The prevalence of the autonomic features for all craniofacial neuralgias in the descending order is as follows, lacrimation, conjunctival injection, nasal congestion, rhinorrhea, flushing, edema/swelling, salivation, ptosis and sweating. With trigeminal neuralgia, the most common autonomic feature was lacrimation, and the least common was nasal congestion. We also found that the intensity and chronicity of the pain were associated with increased autonomic symptoms.

## 6. Clinical implications

• Autonomic features are an important parameter that is associated with the intensity and severity of the pain experience in craniofacial neuralgias.

• Autonomic features profoundly affect the management and the outcome of craniofacial neuralgias.

• Since approximately 40% of trigeminal neuralgia patients present with autonomic features that may affect treatment planning, the astute clinician should carefully look for these features in craniofacial neuralgias.

• Since approximately 30% of craniofacial neuralgias occur with lacrimation as an autonomic feature, it is important for the clinician to attempt to distinguish between a neuralgia with autonomic feature, as opposed to the patient tearing up in pain.

## Supplementary Material


