# Supplementary material for: Autonomic features of craniofacial neuralgias: a systematic review with meta-analysis
Source: J Oral Facial Pain Headache. 2024 Sep 12;38(3):15–31. doi: 10.22514/jofph.2024.023 (PMC11810650; doi:10.22514/jofph.2024.023)
Supplement: Supplementary file 1 [file Supplementary-material.docx]

Supplementary material

Supplementary Table 1. Mesh terms and keywords.

| PubMed | ((“facial neuralgia”[MeSH Terms] OR (“facial”[All Fields] AND “neuralgia”[All Fields]) OR “facial neuralgia”[All Fields] OR (“sphenopalatine”[All Fields] AND “neuralgia”[All Fields]) OR “sphenopalatine neuralgia”[All Fields] OR (“spheno-palatine”[All Fields] AND (“neuralgia”[MeSH Terms] OR “neuralgia”[All Fields] OR “neuralgias”[All Fields])) OR (“facial neuralgia”[MeSH Terms] OR (“facial”[All Fields] AND “neuralgia”[All Fields]) OR “facial neuralgia”[All Fields] OR (“facial”[All Fields] AND “neuralgias”[All Fields]) OR “facial neuralgias”[All Fields]) OR (“facial neuralgia”[MeSH Terms] OR (“facial”[All Fields] AND “neuralgia”[All Fields]) OR “facial neuralgia”[All Fields]) OR ((“cranially”[All Fields] OR “skull”[MeSH Terms] OR “skull”[All Fields] OR “cranial”[All Fields]) AND (“neuralgia”[MeSH Terms] OR “neuralgia”[All Fields] OR “neuralgias”[All Fields])) OR ((“cranially”[All Fields] OR “skull”[MeSH Terms] OR “skull”[All Fields] OR “cranial”[All Fields]) AND (“neuralgia”[MeSH Terms] OR “neuralgia”[All Fields] OR “neuralgias”[All Fields])) OR (“trigeminal neuralgia”[MeSH Terms] OR (“trigeminal”[All Fields] AND “neuralgia”[All Fields]) OR “trigeminal neuralgia”[All Fields]) OR ((“occipital”[All Fields] OR “occipitally”[All Fields] OR “occipitals”[All Fields]) AND (“neuralgia”[MeSH Terms] OR “neuralgia”[All Fields] OR “neuralgias”[All Fields])) OR (“C2-C3”[All Fields] AND (“neuralgia”[MeSH Terms] OR “neuralgia”[All Fields] OR “neuralgias”[All Fields])) OR (“greater”[All Fields] AND (“occipital”[All Fields] OR “occipitally”[All Fields] OR “occipitals”[All Fields]) AND (“neuralgia”[MeSH Terms] OR “neuralgia”[All Fields] OR “neuralgias”[All Fields])) OR (“lesser”[All Fields] AND (“occipital”[All Fields] OR “occipitally”[All Fields] OR “occipitals”[All Fields]) AND (“neuralgia”[MeSH Terms] OR “neuralgia”[All Fields] OR “neuralgias”[All Fields])) OR (“glossopharyngeal nerve diseases”[MeSH Terms] OR (“glossopharyngeal”[All Fields] AND “nerve”[All Fields] AND “diseases”[All Fields]) OR “glossopharyngeal nerve diseases”[All Fields] OR (“glossopharyngeal”[All Fields] AND “neuralgia”[All Fields]) OR “glossopharyngeal neuralgia”[All Fields]) OR (“glosso-pharyngeal”[All Fields] AND (“neuralgia”[MeSH Terms] OR “neuralgia”[All Fields] OR “neuralgias”[All Fields])) OR (“supra-trochlear”[All Fields] AND (“neuralgia”[MeSH Terms] OR “neuralgia”[All Fields] OR “neuralgias”[All Fields])) OR (“supratrochlear”[All Fields] AND (“neuralgia”[MeSH Terms] OR “neuralgia”[All Fields] OR “neuralgias”[All Fields])) OR (“supra”[All Fields] AND “trochlear”[All Fields] AND (“neuralgia”[MeSH Terms] OR “neuralgia”[All Fields] OR “neuralgias”[All Fields])) OR (“supra-orbital”[All Fields] AND (“neuralgia”[MeSH Terms] OR “neuralgia”[All Fields] OR “neuralgias”[All Fields])) OR (“supra”[All Fields] AND (“orbit”[MeSH Terms] OR “orbit”[All Fields] OR “orbits”[All Fields] OR “orbit s”[All Fields] OR “orbital”[All Fields] OR “orbital s”[All Fields] OR “orbitals”[All Fields] OR “orbited”[All Fields] OR “orbiter”[All Fields] OR “orbiters”[All Fields] OR “orbiting”[All Fields]) AND (“neuralgia”[MeSH Terms] OR “neuralgia”[All Fields] OR “neuralgias”[All Fields])) OR (“neuralgia”[MeSH Terms] OR “neuralgia”[All Fields] OR (“supraorbital”[All Fields] AND “neuralgia”[All Fields]) OR “supraorbital neuralgia”[All Fields]) OR (“infratrochlear”[All Fields] AND (“neuralgia”[MeSH Terms] OR “neuralgia”[All Fields] OR “neuralgias”[All Fields])) OR (“infra”[All Fields] AND “trochlear”[All Fields] AND (“neuralgia”[MeSH Terms] OR “neuralgia”[All Fields] OR “neuralgias”[All Fields])) OR (“infra-trochlear”[All Fields] AND (“neuralgia”[MeSH Terms] OR “neuralgia”[All Fields] OR “neuralgias”[All Fields])) OR (“infra”[All Fields] AND (“orbit”[MeSH Terms] OR “orbit”[All Fields] OR “orbits”[All Fields] OR “orbit s”[All Fields] OR “orbital”[All Fields] OR “orbital s”[All Fields] OR “orbitals”[All Fields] OR “orbited”[All Fields] OR “orbiter”[All Fields] OR “orbiters”[All Fields] OR “orbiting”[All Fields]) AND (“neuralgia”[MeSH Terms] OR “neuralgia”[All Fields] OR “neuralgias”[All Fields])) OR ((“infraorbital”[All Fields] OR “infraorbitals”[All Fields]) AND (“neuralgia”[MeSH Terms] OR “neuralgia”[All Fields] OR “neuralgias”[All Fields])) OR (“infra-orbital”[All Fields] AND (“neuralgia”[MeSH Terms] OR “neuralgia”[All Fields] OR “neuralgias”[All Fields])) OR ((“facial nerve”[MeSH Terms] OR (“facial”[All Fields] AND “nerve”[All Fields]) OR “facial nerve”[All Fields] OR (“nervus”[All Fields] AND “intermedius”[All Fields]) OR “nervus intermedius”[All Fields]) AND (“neuralgia”[MeSH Terms] OR “neuralgia”[All Fields] OR “neuralgias”[All Fields])) OR ((“anxiety”[MeSH Terms] OR “anxiety”[All Fields] OR “nervous”[All Fields]) AND “intermedius”[All Fields] AND (“neuralgia”[MeSH Terms] OR “neuralgia”[All Fields] OR “neuralgias”[All Fields])) OR (“auriculotemporal”[All Fields] AND (“neuralgia”[MeSH Terms] OR “neuralgia”[All Fields] OR “neuralgias”[All Fields])) OR ((“facial nerve”[MeSH Terms] OR (“facial”[All Fields] AND “nerve”[All Fields]) OR “facial nerve”[All Fields] OR (“intermediate”[All Fields] AND “nerve”[All Fields]) OR “intermediate nerve”[All Fields]) AND (“wrisberg”[All Fields] OR “wrisberg s”[All Fields]) AND (“neuralgia”[MeSH Terms] OR “neuralgia”[All Fields] OR “neuralgias”[All Fields])) OR ((“great”[All Fields] OR “greats”[All Fields]) AND “auricular”[All Fields] AND (“neuralgia”[MeSH Terms] OR “neuralgia”[All Fields] OR “neuralgias”[All Fields])) OR (“greater”[All Fields] AND “auricular”[All Fields] AND (“neuralgia”[MeSH Terms] OR “neuralgia”[All Fields] OR “neuralgias”[All Fields])) OR (“auricular”[All Fields] AND (“neuralgia”[MeSH Terms] OR “neuralgia”[All Fields] OR “neuralgias”[All Fields])) OR ((“lacrimal”[All Fields] OR “lacrimation”[All Fields]) AND (“neuralgia”[MeSH Terms] OR “neuralgia”[All Fields] OR “neuralgias”[All Fields]))) AND (((“conjunctiva”[MeSH Terms] OR “conjunctiva”[All Fields] OR “conjunctival”[All Fields] OR “conjunctivalization”[All Fields] OR “conjunctivalized”[All Fields]) AND (“inject”[All Fields] OR “injectability”[All Fields] OR “injectant”[All Fields] OR “injectants”[All Fields] OR “injectate”[All Fields] OR “injectates”[All Fields] OR “injected”[All Fields] OR “injectible”[All Fields] OR “injectibles”[All Fields] OR “injecting”[All Fields] OR “injections”[MeSH Terms] OR “injections”[All Fields] OR “injectable”[All Fields] OR “injectables”[All Fields] OR “injection”[All Fields] OR “injects”[All Fields])) OR (“prolapse”[MeSH Terms] OR “prolapse”[All Fields] OR “ptosis”[All Fields] OR “blepharoptosis”[MeSH Terms] OR “blepharoptosis”[All Fields]) OR (“miosis”[MeSH Terms] OR “miosis”[All Fields]) OR (“mydriasis”[MeSH Terms] OR “mydriasis”[All Fields]) OR (“tearings”[All Fields] OR “tears”[MeSH Terms] OR “tears”[All Fields] OR “tearing”[All Fields] OR “lacrimal apparatus diseases”[MeSH Terms] OR (“lacrimal”[All Fields] AND “apparatus”[All Fields] AND “diseases”[All Fields]) OR “lacrimal apparatus diseases”[All Fields] OR “lacerations”[MeSH Terms] OR “lacerations”[All Fields]) OR (“lacrimal”[All Fields] OR “lacrimation”[All Fields]) OR (“congested”[All Fields] OR “congestion”[All Fields] OR “congestions”[All Fields] OR “congestive”[All Fields]) OR (“rhinorrhea”[MeSH Terms] OR “rhinorrhea”[All Fields] OR “rhinorrhoea”[All Fields] OR “rhinorrheas”[All Fields]) OR (“salivation”[MeSH Terms] OR “salivation”[All Fields] OR “salivate”[All Fields] OR “salivated”[All Fields] OR “salivating”[All Fields]) OR (“flushing”[MeSH Terms] OR “flushing”[All Fields] OR “flush”[All Fields] OR “flushed”[All Fields] OR “flushes”[All Fields] OR “blushing”[MeSH Terms] OR “blushing”[All Fields] OR “flushings”[All Fields]) OR (“sweat”[MeSH Terms] OR “sweat”[All Fields] OR “sweating”[MeSH Terms] OR “sweating”[All Fields] OR “sweats”[All Fields] OR “sweatings”[All Fields]) OR (“hyperhidrosis”[MeSH Terms] OR “hyperhidrosis”[All Fields]) OR (“edema”[MeSH Terms] OR “edema”[All Fields] OR “swelling”[All Fields] OR “swell”[All Fields] OR “swelled”[All Fields] OR “swellings”[All Fields] OR “swells”[All Fields]) OR (“edema”[MeSH Terms] OR “edema”[All Fields] OR “edemas”[All Fields] OR “oedemas”[All Fields] OR “oedema”[All Fields]) OR “red”[All Fields] OR (“erythema”[MeSH Terms] OR “erythema”[All Fields] OR “redness”[All Fields]) OR (“autonomic nervous system”[MeSH Terms] OR (“autonomic”[All Fields] AND “nervous”[All Fields] AND “system”[All Fields]) OR “autonomic nervous system”[All Fields] OR “autonomic”[All Fields] OR “autonomical”[All Fields] OR “autonomically”[All Fields] OR “autonomics”[All Fields]) OR ((“autonomic nervous system”[MeSH Terms] OR (“autonomic”[All Fields] AND “nervous”[All Fields] AND “system”[All Fields]) OR “autonomic nervous system”[All Fields] OR “autonomic”[All Fields] OR “autonomical”[All Fields] OR “autonomically”[All Fields] OR “autonomics”[All Fields]) AND (“diagnosis”[MeSH Subheading] OR “diagnosis”[All Fields] OR “symptoms”[All Fields] OR “diagnosis”[MeSH Terms] OR “symptom”[All Fields] OR “symptom s”[All Fields] OR “symptomes”[All Fields])) OR (“sympathetic”[All Fields] OR “sympathetically”[All Fields] OR “sympathetics”[All Fields]) OR (“parasympathetic”[All Fields] OR “parasympathetically”[All Fields] OR “parasympathetics”[All Fields])) AND (“humans”[MeSH Terms] AND “english”[Language] AND 1990/01/01:2022/09/30[Date - Publication])) NOT (“Review”[Publication Type] OR “review literature as topic”[MeSH Terms] OR “Review”[All Fields] OR (“meta analysis”[Publication Type] OR “meta analysis as topic”[MeSH Terms] OR “meta analysis”[All Fields]) OR “systematic review”[Publication Type]) |
| --- | --- |
| Embase | SourcesEmbase, Embase Classic, MEDLINE, Preprints  Query(sphenopalatine AND (‘neuralgia’/exp OR neuralgia) OR (‘spheno palatine’ AND neuralgia) OR (facial AND neuralgias) OR (facial AND neuralgia) OR (cranial AND neuralgia) OR (cranial AND neuralgias) OR (trigeminal AND neuralgia) OR (occipital AND neuralgia) OR (‘c2 c3’ AND neuralgia) OR (greater AND occipital AND neuralgia) OR (lesser AND occipital AND neuralgia) OR (glossopharyngeal AND neuralgia) OR (‘glosso pharyngeal’ AND neuralgia) OR (‘supra trochlear’ AND neuralgia) OR (supratrochlear AND neuralgia) OR (supra AND trochlear AND neuralgia) OR (‘supra orbital’ AND neuralgia) OR (supra AND orbital AND neuralgia) OR (supraorbital AND neuralgia) OR (infratrochlear AND neuralgia) OR (infra AND trochlear AND neuralgia) OR (‘infra trochlear’ AND neuralgia) OR (infra AND orbital AND neuralgia) OR (infraorbital AND neuralgia) OR (‘infra orbital’ AND neuralgia) OR (nervus AND intermedius AND neuralgia) OR (nervous AND intermedius AND neuralgia) OR (auriculotemporal AND neuralgia) OR (intermediate AND nerve AND of AND wrisberg AND neuralgia) OR (great AND auricular AND neuralgia) OR (greater AND auricular AND neuralgia) OR (auricular AND neuralgia) OR (lacrimal AND neuralgia)) AND (conjunctival AND injection OR ptosis OR miosis OR mydriasis OR tearing OR lacrimation OR congestion OR rhinorrhea OR salivation OR flushing OR sweating OR hyperhidrosis OR swelling OR edema OR red OR redness OR autonomic OR (autonomic AND symptoms) OR sympathetic OR parasympathetic) AND [article]/lim AND [humans]/lim AND [1990-2022]/py AND [english]/lim. |
| Ovid MEDLINE | Ovid MEDLINE(R) <1946 to 30 September 2022>  1. (sphenopalatine neuralgia or spheno-palatine neuralgia or facial neuralgias or facial neuralgia or cranial neuralgias or trigeminal neuralgia or occipital neuralgia or C2–C3 neuralgia or greater occipital neuralgia or lesser occipital neuralgia or glossopharyngeal neuralgia or glosso-pharyngeal neuralgia or supra-trochlear neuralgia or supratrochlear neuralgia or supra trochlear neuralgia or supra-orbital neuralgia or supra orbital neuralgia or supraorbital neuralgia or infratrochlear neuralgia or infra trochlear neuralgia or infra-trochlear neuralgia or infra orbital neuralgia or infraorbital neuralgia or infra-orbital neuralgia or nervus intermedius neuralgia or nervous intermedius neuralgia or auriculotemporal neuralgia or intermediate nerve of Wrisberg neuralgia or great auricular neuralgia or greater auricular neuralgia or auricular neuralgia or lacrimal neuralgia).mp. [mp = title, abstract, original title, name of substance word, subject heading word, floating sub-heading word, keyword heading word, organism supplementary concept word, protocol supplementary concept word, rare disease supplementary concept word, unique identifier, synonyms]  2. (conjunctival injection or ptosis or miosis or mydriasis or tearing or lacrimation or congestion or rhinorrhea or salivation or flushing or sweating or hyperhidrosis or swelling or edema or red or redness or autonomic or autonomic symptoms or sympathetic or parasympathetic).mp. [mp = title, abstract, original title, name of substance word, subject heading word, floating sub-heading word, keyword heading word, organism supplementary concept word, protocol supplementary concept word, rare disease supplementary concept word, unique identifier, synonyms]  3. 1 and 2  4. limit 3 to (english language and humans and yr = “1990-Current”) |
| Scopus | ((TITLE-ABS-KEY (conjunctival AND injection) OR TITLE-ABS-KEY (ptosis) OR TITLE-ABS-KEY (miosis) OR TITLE-ABS-KEY (tearing) OR TITLE-ABS-KEY (lacrimation) OR TITLE-ABS-KEY (congestion) OR TITLE-ABS-KEY (rhinorrhea) OR TITLE-ABS-KEY (salivation) OR TITLE-ABS-KEY (flushing) OR TITLE-ABS-KEY (sweating) OR TITLE-ABS-KEY (hyperhidrosis) OR TITLE-ABS-KEY (swelling) OR TITLE-ABS-KEY (edema) OR TITLE-ABS-KEY ( red ) OR TITLE-ABS-KEY (redness) OR TITLE-ABS-KEY (autonomic) OR TITLE-ABS-KEY (autonomic AND symptoms) OR TITLE-ABS-KEY (sympathetic) OR TITLE-ABS-KEY (parasympathetic)) AND PUBYEAR > 1989) AND ((TITLE-ABS-KEY (sphenopalatine AND neuralgia) OR TITLE-ABS-KEY (facial AND neuralgia) OR TITLE-ABS-KEY (cranial AND neuralgia) OR TITLE-ABS-KEY (trigeminal AND neuralgia) OR TITLE-ABS-KEY (occipital AND neuralgia) OR TITLE-ABS-KEY (c2–c3 AND neuralgia) OR TITLE-ABS-KEY (glossopharyngeal AND neuralgia) OR TITLE-ABS-KEY (supra AND trochlear AND neuralgia) OR TITLE-ABS-KEY (supraorbital AND neuralgia) OR TITLE-ABS-KEY (infratrochlear AND neuralgia) OR TITLE-ABS-KEY (infraorbital AND neuralgia) OR TITLE-ABS-KEY (nervus AND intermedius AND neuralgia) OR TITLE-ABS-KEY (auriculotemporal AND neuralgia) OR TITLE-ABS-KEY (intermediate AND nerve AND of AND wrisberg AND neuralgia) OR TITLE-ABS-KEY (auricular AND neuralgia) OR TITLE-ABS-KEY (lacrimal AND neuralgia)) AND PUBYEAR >1989 ) AND (LIMIT-TO (DOCTYPE, “ar”)) AND (LIMIT-TO (LANGUAGE, “English”)) |
| Web of science | #1 conjunctival injection (All Fields) or ptosis (All Fields) or miosis (All Fields) or mydriasis (All Fields) or tearing (All Fields) or lacrimation (All Fields) or congestion (All Fields) or rhinorrhea (All Fields) or salivation (All Fields) or flushing (All Fields) or sweating (All Fields) or hyperhidrosis (All Fields) or swelling (All Fields) or edema (All Fields) or red (All Fields) or redness (All Fields) or autonomic (All Fields) or autonomic symptoms (All Fields) or sympathetic (All Fields) or parasympathetic (All Fields)  #2 ALL=(sphenopalatine neuralgia) OR ALL=(facial neuralgia) OR ALL=(cranial neuralgia) OR ALL=(cranial neuralgias) OR ALL=(trigeminal neuralgia) OR ALL=(occipital neuralgia) OR ALL=(C2–C3 neuralgia) OR ALL=(greater occipital neuralgia) OR ALL=(lesser occipital neuralgia) OR ALL=(glossopharyngeal neuralgia) OR ALL=(glosso-pharyngeal neuralgia) OR ALL=(supra-trochlear neuralgia) OR ALL=(supratrochlear neuralgia) OR ALL=(supra trochlear neuralgia) OR ALL=(supra-orbital neuralgia) OR ALL=(supra orbital neuralgia) OR ALL=(supraorbital neuralgia) OR ALL=(infracochlear neuralgia) OR ALL=(infra trochlear neuralgia) OR ALL=(infra-trochlear neuralgia) OR ALL=(infra orbital neuralgia) OR ALL=(infraorbital neuralgia) OR ALL=(infra-orbital neuralgia) OR ALL=(nervus intermedius neuralgia) OR ALL=(nervous intermedius neuralgia) OR ALL=(auriculotemporal neuralgia) OR ALL=(intermediate nerve of weisberg neuralgia) OR ALL=(great auricular neuralgia) OR ALL=(greater auricular neuralgia) OR ALL=(auricular neuralgia) OR ALL=(lacrimal neuralgia)  (#1) AND #2 and 2022 or 2021 or 2020 or 2019 or 2018 or 2017 or 2016 or 2015 or 2014 or 2013 or 2012 or 2011 or 2010 or 2009 or 2008 or 2007 or 2006 or 2005 or 2004 or 2002 or 2001 or 2003 or 2000 or 1999 or 1998 or 1997 or 1996 or 1995 or 1994 or 1993 or 1992 or 1991 or 1990 (Publication Years) and Articles (Document Types) |

Supplementary Table 1-1. Joanna Brigg’s institute critical appraisal tool for case report.

| Author & year | Type of study | Q1 | Q2 | Q3 | Q4 | Q5 | Q6 | Q7 | Q8 | Overall, ROB |
| --- | --- | --- | --- | --- | --- | --- | --- | --- | --- | --- |
| Bouhassira, 1994 | Case report | Yes | Yes | Yes | Yes | Yes | No | No | Yes | Low |
| Sesso, 2001 | Case report | Yes | Yes | Yes | Yes | Yes | Yes | Yes | No | Low |
| Pareja, 2002 | Case report | Unclear | No | Yes | Yes | Yes | Yes | Yes | Yes | Low |
| Sato, 2007 | Case report | Unclear | Yes | Yes | Yes | Yes | Yes | Yes | Unclear | Low |
| Riederer, 2010 | Case report | No | Yes | Yes | Yes | Unclear | Unclear | Yes | Yes | Low |
| Pareja, 2013 | Case report | No | Yes | Yes | Yes | Yes | Yes | Yes | Yes | Low |
| Khan, 2015 | Case report | Yes | Yes | Yes | Yes | Yes | Yes | Unclear | Yes | Low |
| Pirillo, 2018 | Case report | No | No | Unclear | Unclear | Yes | Yes | No | Yes | High |
| Lee, 2019 | Case report | Unclear | Yes | Yes | Yes | Yes | No | Yes | Yes | Low |
| Onoda, 2020 | Case report | Yes | Yes | Yes | Yes | Yes | Yes | Yes | Yes | Low |
| Thomas, 2021 | Case report | Yes | Unclear | Yes | Yes | Yes | Yes | Yes | Yes | Low |

Supplementary Table 1-2. Joanna Brigg’s institute critical appraisal tool for case series.

| Author & year | Type of study | Q1 | Q2 | Q3 | Q4 | Q5 | Q6 | Q7 | Q8 | Q9 | Q10 | Overall, ROB |
| --- | --- | --- | --- | --- | --- | --- | --- | --- | --- | --- | --- | --- |
| Pareja, 2015 | Case series | Unclear | Yes | Yes | Unclear | Yes | Yes | Yes | Yes | Unclear | NA | Low |
| Pareja, 2017 | Case series | Unclear | Yes | Yes | Yes | No | Yes | Yes | Yes | Yes | NA | Low |
| Villar-Quiles, 2018 | Case series | Yes | Yes | Yes | No | No | Yes | Yes | Yes | Unclear | NA | High |

Supplementary Table 1-3. Joanna Brigg’s institute critical appraisal tool for cross-sectional studies.

| Author & year | Type of study | Q1 | Q2 | Q3 | Q4 | Q5 | Q6 | Q7 | Q8 | Overall, ROB |
| --- | --- | --- | --- | --- | --- | --- | --- | --- | --- | --- |
| Sjaastad, 1997 | Cross-sectional | Yes | Unclear | Yes | Yes | No | No | Yes | Yes | Unclear |
| Benoliel, 1998 | Cross-sectional | Yes | Yes | Unclear | Yes | Unclear | Unclear | Yes | NA | Unclear |
| Simms, 2011 | Cross-sectional | Unclear | Yes | NA | Yes | NA | NA | Yes | Yes | No information |
| Molina, 2014 | Cross-sectional | Yes | No | Unclear | No | No | No | Yes | Yes | High |
| Maarjberg, 2014 | Cross-sectional | Yes | Yes | Yes | Yes | NA | NA | Yes | Yes | Low |

Supplementary Table 1-4. Joanna Brigg’s institute critical appraisal tool for cohort studies.

| Author & year | Type of study | Q1 | Q2 | Q3 | Q4 | Q5 | Q6 | Q7 | Q8 | Q9 | Q10 | Q11 | Overall, ROB |
| --- | --- | --- | --- | --- | --- | --- | --- | --- | --- | --- | --- | --- | --- |
| Rasmussen, 1991 | Cohort study | Yes | Yes | Yes | Unclear | Unclear | No | NA | Yes | Yes | NA | No | Unclear |
| Benoliel, 2009 | Cohort study | Yes | Yes | Yes | Unclear | Unclear | Yes | Yes | Yes | Yes | Yes | Yes | Low |
| Haviv, 2015 | Cohort study | Yes | Yes | Yes | Unclear | Unclear | Yes | Yes | Yes | Yes | Yes | Yes | Low |


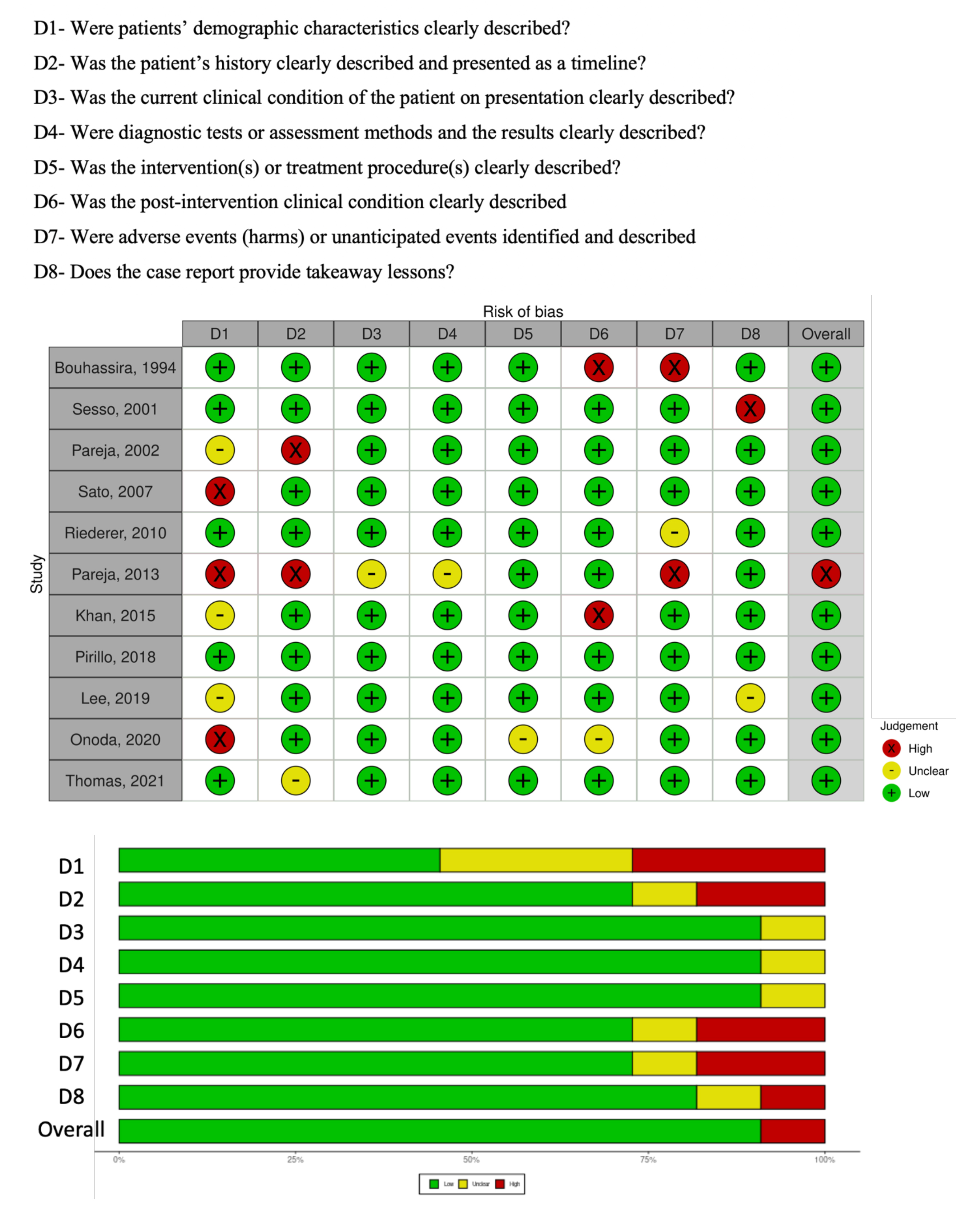


Supplementary Fig. 1. Joanna Brigg’s institute critical appraisal tool for case report.


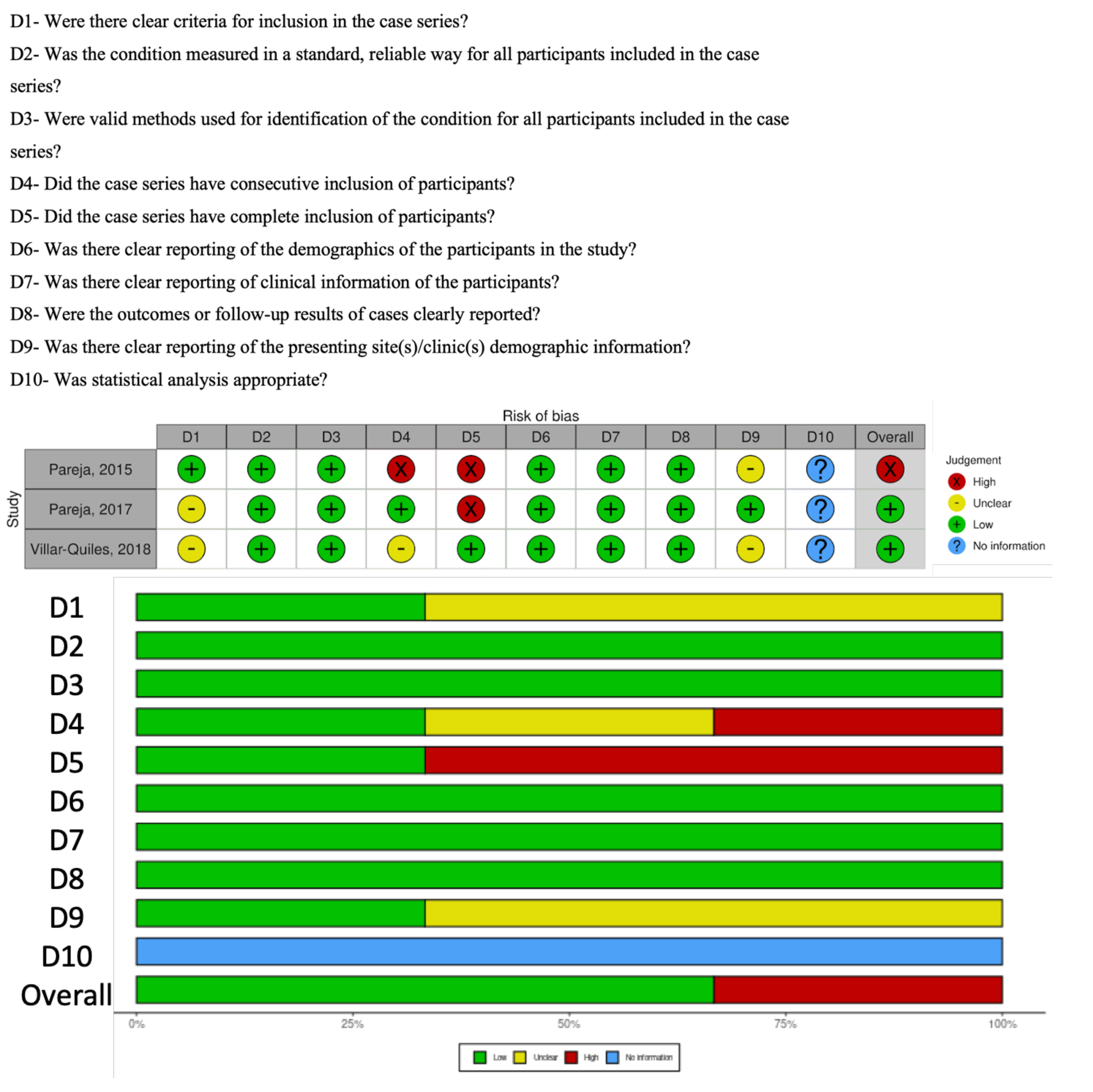


Supplementary Fig. 2. Joanna Brigg’s institute critical appraisal tool for case series.


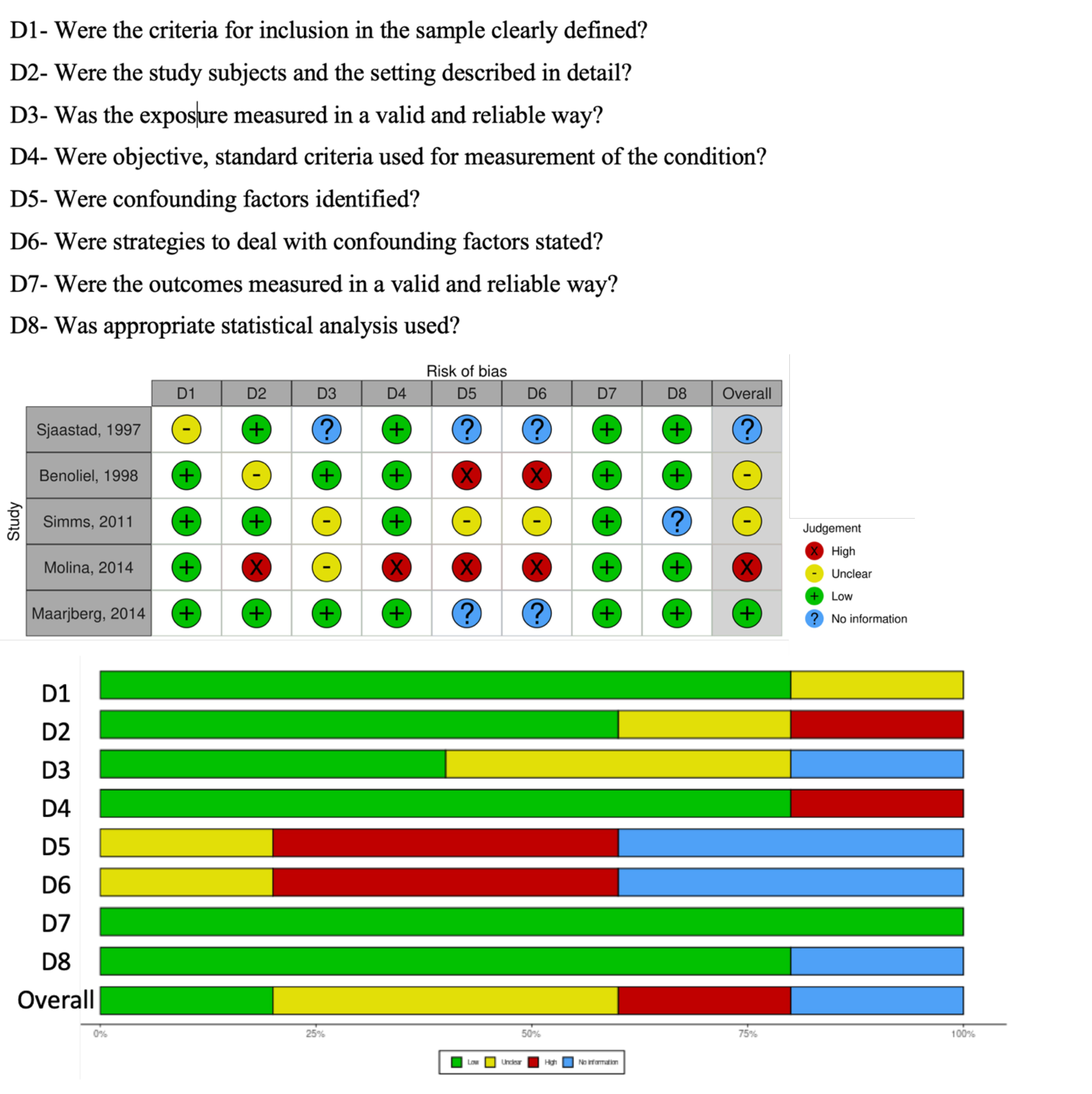


Supplementary Fig. 3. Joanna Brigg’s institute critical appraisal tool for cross-sectional studies.


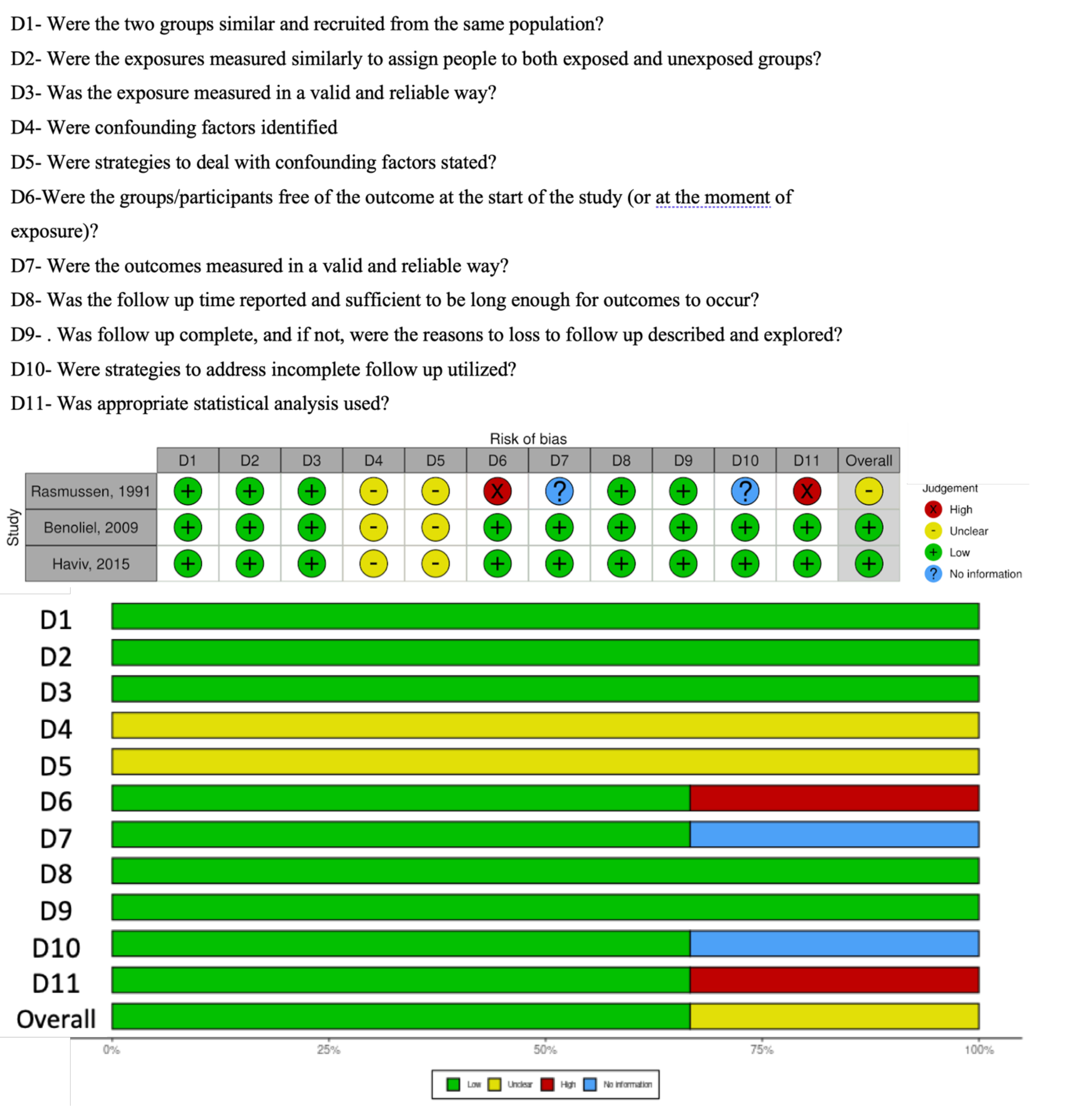


Supplementary Fig. 4. Joanna Brigg’s institute critical appraisal tool for cohort studies.
